# Differential Role of PD-1 Expressed by Various Immune and Tumor Cells in the Tumor Immune Microenvironment: Expression, Function, Therapeutic Efficacy, and Resistance to Cancer Immunotherapy

**DOI:** 10.3389/fcell.2021.767466

**Published:** 2021-11-22

**Authors:** Myeong Joon Kim, Sang-Jun Ha

**Affiliations:** ^1^ Department of Biochemistry, College of Life Science and Biotechnology, Yonsei University, Seoul, South Korea; ^2^ Brain Korea 21 (BK21) FOUR Program, Yonsei Education & Research Center for Biosystems, Yonsei University, Seoul, South Korea

**Keywords:** tumor microenvironment, cancer immunotherapy, programmed cell death protein 1 (PD-1), tumor-infiltrating effector cells, tumor-infiltrating suppressive cells, functional restoration

## Abstract

In the tumor immune microenvironment (TIME), tumor cells interact with various cells and operate various strategies to avoid antitumor immune responses. These immune escape strategies often make the TIME resistant to cancer immunotherapy. Neutralizing immune escape strategies is necessary to overcome resistance to cancer immunotherapy. Immune checkpoint receptors (ICRs) expressed in effector immune cells inhibit their effector function via direct interaction with immune checkpoint ligands (ICLs) expressed in tumor cells. Therefore, blocking ICRs or ICLs has been developed as a promising cancer immunotherapy by reinvigorating the function of effector immune cells. Among the ICRs, programmed cell death 1 (PD-1) has mainly been antagonized to enhance the survival of human patients with cancer by restoring the function of tumor-infiltrating (TI) CD8^+^ T cells. It has been demonstrated that PD-1 is expressed not only in TI CD8^+^ T cells, but also in other TI immune cells and even tumor cells. While PD-1 suppresses the function of TI CD8^+^ T cells, it is controversial whether PD-1 suppresses or amplifies the suppressive function of TI-suppressive immune cells (e.g., regulatory T cells, tumor-associated macrophages, and myeloid cells). There is also controversy regarding the role of tumor-expressing PD-1. Therefore, a precise understanding of the expression pattern and function of PD-1 in each cell subset is important for improving the efficacy of cancer immunotherapy. Here, we review the differential role of PD-1 expressed by various TI immune cells and tumor cells. We focused on how cell-type-specific ablation or blockade of PD-1 affects tumor growth in a murine tumor model. Furthermore, we will also describe how the blockade of PD-1 acts on TI immune cells in human patients with cancer.

## 1 Introduction

CD8^+^ T cells in the TIME are exposed to chronic antigen stimulation (Wherry and Kurachi, 2015). Chronic antigen stimulation gradually leads CD8^+^ T cells to an exhausted state (Wherry and Kurachi, 2015). The exhausted CD8^+^ T cells have distinct characteristics compared to effector CD8^+^ T cells (Wherry and Kurachi, 2015). First, exhausted CD8^+^ T cells express a variety of immune checkpoint receptors (ICRs), including programmed cell death 1 (PD-1), T cell immunoglobulin and mucin-domain containing-3 (TIM3), lymphocyte activation gene 3 protein (LAG3), and T cell immunoreceptor with Ig and ITIM domains (TIGIT) (Wherry and Kurachi, 2015; Anderson et al., 2016; Zelba et al., 2019). ICRs transduce inhibitory signals into exhausted CD8^+^ T cells (McLane et al., 2019). Among various ICRs, exhausted CD8^+^ T cells express high levels of PD-1. Second, exhausted CD8^+^ T cells are transcriptionally altered (Khan et al., 2019). Various transcription factors responsible for T cell exhaustion (e.g., Eomes, TOX, and Blimp1) are expressed in exhausted CD8^+^ T cells (Shin et al., 2009; Buggert et al., 2014; Wang et al., 2019a; Khan et al., 2019; McLane et al., 2019; Kim et al., 2020; Han et al., 2021). Eventually, exhausted CD8^+^ T cells are unable to respond to tumor cells. As functional restoration of exhausted CD8^+^ T cells is important for effective antitumor immunity, advanced analytic tools (e.g., transposase-accessible chromatin using sequencing (ATAC-seq) and single-cell RNA sequencing (scRNA-seq)) have been applied to identify the epigenetic characteristics and transcriptomes of exhausted CD8^+^ T cells to improve our understanding of cancer immunotherapy (Thommen et al., 2018; Wang et al., 2019a; Khan et al., 2019; Kim et al., 2020). Interestingly, it has been revealed that exhaustion also occurs in other immune cells (e.g., CD4^+^ T cells, and TAMs) and that high PD-1 expression is strongly associated with exhaustion in all cell types (Wherry and Kurachi, 2015; Zha et al., 2021).

Cancer immunotherapy using anti-PD-1 antibodies (PD-1 therapy) has been thought to enhance antitumor immunity by reinvigorating the functionality of tumor-infiltrating (TI) PD-1^+^CD8^+^ T cells (Wherry and Kurachi, 2015; Thommen et al., 2018; Kim K. H. et al., 2019). Recently, it has been demonstrated that PD-1 is also expressed on other cells (e.g., Tregs, TAMs, and tumor cells) and that PD-1 therapy enhances antitumor immunity in a diverse cell-dependent manner (Karyampudi et al., 2016; Xiao et al., 2016; Gordon et al., 2017; Yao et al., 2018; Moral et al., 2020; Strauss et al., 2020; Zhang and Liu, 2020; Lim et al., 2021; Zha et al., 2021). PD-1 in effector immune cells mainly inhibits their effector function and promotes tumor progression (Gordon et al., 2017; Zhang and Liu, 2020; Zha et al., 2021). However, the function of PD-1 in some suppressive immune cells and tumor cells has been controversial (Kleffel et al., 2015; Lowther et al., 2016; Stathopoulou et al., 2018; Kim H. R. et al., 2019; Kamada et al., 2019; Wang X. et al., 2020; Kumagai et al., 2020; Yoshida et al., 2020; Lim et al., 2021). This unclear function of PD-1 in specific cell types makes it difficult to predict the responsiveness of PD-1 therapy. Therefore, an accurate understanding of PD-1 function in each cell type is crucial for successful PD-1 therapy. This review will focus on PD-1 expression in various immune cells and tumor cells in terms of expression, function, therapeutic effect, and resistance to PD-1 therapy.

## 2 CD8^+^ T Cells

CD8^+^ T cells are a key population in the TIME for effective antitumor immunity because CD8^+^ T cells directly kill tumor cells by secreting effector cytokines (e.g., IFN-γ, TNF-α, and IL-2) (Hashimoto et al., 2018). TI CD8^+^ T cells highly express PD-1 (Wherry and Kurachi, 2015; Hashimoto et al., 2018) (Table 1).

**TABLE 1:** PD-1 expressed on tumor-infiltrating immune cells.

Cell types	Expression	Function	Mechanism	References
CD8^+^ T cells	*Positive regulation* TCR engagement, NFAT, AP-1, Foxo1, Notch, and TOX *Negative regulation* FBXO38	Inhibition of CD8^+^ T cell-mediated cytotoxicity and CD8^+^ T cell proliferation. Induction of T cell exhaustion	Inhibition of TCR downstream signaling and CD28 costimulatory signaling	Bally et al. (2016), Hui et al. (2017), Kamphorst et al. (2017b), Khan et al. (2019), Macian et al. (2001), Mathieu et al. (2013), Meng et al. (2018), Oestreich et al. (2008), Staron et al. (2014)
Tconvs		Inhibition of Tconv function (cytokine secretion, DC maturation, and cytotoxicity). Induction of T cell exhaustion	Inhibition of TCR downstream signaling and IL-21 expression	Balanca et al. (2021), Bronsert et al. (2020), Nagasaki et al. (2020), Oh et al. (2020), Sanchez-Alonso et al. (2020), Shi et al. (2018a), Shi et al. (2018b)
Tregs	*Positive regulation* TCR engagement and SREBP signaling	Inhibition of Treg suppressive function and stability	Inhibition of the phosphorylation of AKT and S6	Kamada et al. (2019), Kumagai et al. (2020)
		Amplification of Treg suppressive function and stability	Maintenance of Foxp3 expression by inhibiting AEP. Maintenance of lipid metabolism by inhibiting the activation of PI3K and the phosphorylation of S6 and AKT	Lim et al. (2021), Stathopoulou et al. (2018), Yoshida et al. (2020)
B cells	*Positive regulation* CD40 signaling, JNK, p38, NF-κB, and Bcl6 *Negative regulation* IL-4 signaling	Induction of IL-10 expression (human advanced-stage hepatocellular carcinoma)	Mechanism was not specified	Wang et al. (2019b), Xiao et al. (2016)
NK cells	*Positive regulation* GCs, IL-12, IL-15, and IL-18	Inhibition of NK cell-mediated cytotoxicity	Inhibition of the activation of PI3K/AKT signaling	Concha-Benavente et al. (2018), Liu et al. (2017), Quatrini et al. (2021)
ILCs	*Positive regulation* IL-2, IL-7, and IL-33 (ILC2)	Inhibition of expression of ILC2 effector molecules and CD103^+^ DC-mediated CD8^+^ T cell activation	Mechanism was not specified	Moral et al. (2020), Taylor et al. (2017), Wang et al. (2020b)
TAMs	*Positive regulation* Type I IFN, NF-κB, TLR2/4 agonist, and MyD88/IL-1R axis *Negative regulation* c-Cbl	Inhibition of phagocytosis. Induction of M1 to M2 transition	Mechanism was not specified	Dhupkar et al. (2018), Gordon et al. (2017), Kono et al. (2020), Rao et al. (2020)
DCs	*Positive regulation* IL-10	Inhibition of cytokine secretion, costimulatory molecules expression, antigen presentation, and CD8^+^ T cell function	Inhibition of NF-κB translocation into the nucleus by preventing IκBα degradation	Karyampudi et al. (2016), Krempski et al. (2011), Lamichhane et al. (2017), Lim et al. (2016)
		Induction of T cell activation	PD-L1 blockade by *cis* interaction	Zhao et al. (2018)
Myeloid cells	*Positive regulation* G-CSF, GM-CSF, and TLR4 agonist	Inhibition of glycolysis, pentose phosphate pathway, TCA cycle, and cholesterol synthesis. Generation of MDSCs	Inhibition of ERK1/2, mTORC1, and STAT1 activation	Strauss et al. (2020)

Tconvs, CD4^+^Foxp3^-^ conventional T cells; Tregs, CD4^+^Foxp3^+^ regulatory T cells; NK, cells, natural killer cells; ILCs, innate lymphoid cells; TAMs, tumor-associated macrophages; DCs, dendritic cells. TCR, T cell receptor; NFAT, nuclear factor of activated T cells; AP-1, activator protein 1; TOX, thymocyte selection-associated with high mobility group box protein; FBXO38, f-box protein only protein 38; IL, interleukin; SREBP, sterol regulatory element-binding protein; JNK, c-jun N-terminal kinase; NF-κB, nuclear factor kappa-light-chain-enhancer of activated B cells; Bcl6, B cell lymphoma 6; GC, glucocorticoid; PI3K, phosphoinositide 3-kinase; IFN, interferon; TLR, toll-like receptor; MyD88, myeloid differentiation factor 88; c-Cbl, castias B lymphoma; IκBα, nuclear factor of kappa light polypeptide gene enhancer in B-cells inhibitor, alpha; PD-L1, programmed death-ligand 1; G-CSF, granulocyte colony-stimulating factor; GM-CSF, granulocyte-macrophage colony-stimulating factor; TCA, cycle, tricarboxylic acid cycle; MDSCs, myeloid-derived suppressive cells; ERK, extracellular signal-regulated kinase; mTOR, mammalian target of rapamycin; STAT, signal transducer and activator of transcription.

### 2.1 Expression

In CD4^+^ and CD8^+^ T cells, the mechanism of PD-1 expression is well documented (Macian et al., 2001; Oestreich et al., 2008; Mathieu et al., 2013; Wang et al., 2019a; Khan et al., 2019; Kim et al., 2020; Han et al., 2021). When the T cell receptor (TCR) on CD8^+^ T cells is engaged with the antigen-restricted major histocompatibility complex (MHC) I, CD8^+^ T cells express PD-1 on their surface (Agata et al., 1996). Various transcription factors (e.g., NFAT2, AP-1, Notch, Foxo1, and TOX) have been identified as inducers of PD-1 expression in CD8^+^ T cells upon T cell activation (Macian et al., 2001; Oestreich et al., 2008; Mathieu et al., 2013; Staron et al., 2014; Bally et al., 2016; Khan et al., 2019). Among these transcription factors, TOX is recently identified and emphasized as a major transcription factor responsible for inducing the exhaustion of TI CD8^+^ T cells (Wang et al., 2019a; Khan et al., 2019; Kim et al., 2020; Han et al., 2021). Mechanistically, TOX, induced by NFAT2, regulates the transcriptional and epigenetic effects of exhausted T cells (Khan et al., 2019). PD-1 is downregulated by FBXO38 E3 ligase in a proteasome-dependent manner (Meng et al., 2018). Notably, PD-1 expression in Tregs is unaffected by FBXO38 ablation, while PD-1 expression in CD8^+^ T cells and CD4^+^CD25^−^ T cells is augmented by FBXO38 ablation (Meng et al., 2018), suggesting immune cell type-specific regulation of PD-1 expression.

### 2.2 Function

**FIGURE 1:**
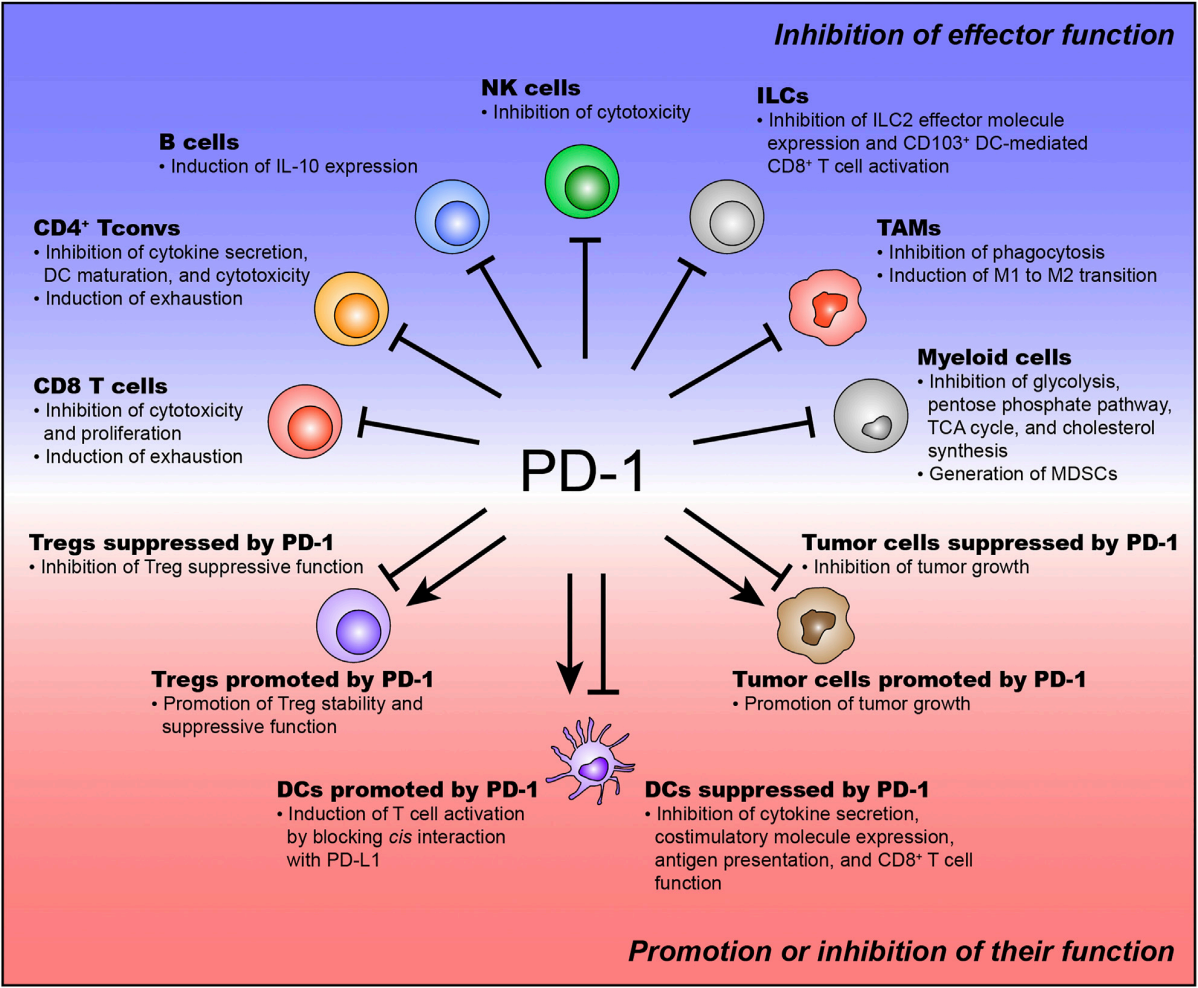
The function of PD-1 expressed on various immune and tumor cells. PD-1 is expressed on various immune and tumor cells. PD-1 expressed on effector immune cells usually inhibits their effector function. The function of PD-1 expressed on suppressive immune cells and tumor cells has been controversial.

PD-1 has been found to inhibit the effector function of CD8^+^ T cells to prevent excessive activation (Sharpe and Pauken, 2018). Mechanistically, PD-1 suppresses various TCR downstream signaling pathways responsible for effector T cell function (e.g., AKT, PI3K, and mTOR) (Riley, 2009; Patsoukis et al., 2012; Pauken and Wherry, 2015; Sharpe and Pauken, 2018). According to this mechanism, TI PD-1^+^CD8^+^ T cells lose their ability to proliferate and produce effector cytokines upon TCR engagement by PD-1 (Pauken and Wherry, 2015; Sharpe and Pauken, 2018). Recently, several studies have demonstrated that PD-1 recruits SHP2 phosphatase and preferentially inhibits CD28 costimulatory signaling rather than TCR signaling (Kamphorst et al., 2017b; Hui et al., 2017; Kim et al., 2021). Kamphorst *et al.* also demonstrate that CD28-deficient T cells is not affected by PD-1 therapy. Additionally, PD-1 signaling regulates transcriptomic and epigenetic programs in TI CD8^+^ T cells by inducing TOX expression (Khan et al., 2019). The chromatin regions that are related to effector T cell differentiation are denied being accessed by TOX (Khan et al., 2019). Meanwhile, the accessibility of genes related to T cell exhaustion is enhanced by TOX (Khan et al., 2019). These results indicate that PD-1 promotes T cell exhaustion and inhibits T cell activation via TOX-induced transcriptional and epigenetic reprogramming (Figure 1). Collectively, PD-1 represses the functionality of TI CD8^+^ T cells by inhibiting TCR/CD28 signaling and regulating transcriptional and epigenetic programs (Figure 1).

### 2.3 Blockade Effect

PD-1 therapy restores the functionality of TI CD8^+^ T cells in various tumor types (Table 2). Mechanistically, as mentioned above, functional restoration of TI PD-1^+^CD8^+^ T cells by PD-1 therapy is dependent on CD28 expression on TI PD-1^+^CD8^+^ T cells (Kamphorst et al., 2017b; Hui et al., 2017; Kim et al., 2021). Therefore, examination of CD28 expression on TI PD-1^+^CD8^+^ T cells can predict the responsiveness of PD-1 therapy in human cancer patients. PD-1 therapy also restores the proliferative capacity of TI PD-1^+^CD8^+^ T cells in the peripheral blood (Kamphorst et al., 2017a; Kim K. H. et al., 2019). This finding suggests that increased Ki67^+^ populations in circulating PD-1^+^CD8^+^ T cells after PD-1 therapy could predict the responsiveness of PD-1 therapy in various tumor types (Kamphorst et al., 2017a; Kim K. H. et al., 2019). Additionally, the ratio of Ki67^+^ population fold change in PD-1^+^CD8^+^ T cells to tumor burden positively correlates with the responsiveness of PD-1 therapy, indicating that pre-existing proliferative TI PD-1^+^CD8^+^ T cell frequency is important in predicting the responsiveness of PD-1 therapy (Huang et al., 2017). Notably, in hepatocellular carcinoma (HCC), PD-1 therapy is recently shown to induce tumor progression (Pfister et al., 2021). Non-alcoholic steatohepatitis (NASH) is a well-known trigger of HCC (Dudek et al., 2021; Pfister et al., 2021). In liver tissue, CXCR6^+^PD-1^+^CD8^+^ T cells are defined as highly auto-aggressive T cells (Dudek et al., 2021; Pfister et al., 2021). Tissue damage induced by auto-aggressive CD8^+^ T cells could lead to the occurrence of HCC in a NASH mouse model (Pfister et al., 2021). They also demonstrate that CD8^+^ T cell depletion in NASH mice reduces the incidence of HCC (Pfister et al., 2021). Furthermore, they identify that PD-1 therapy-induced auto-aggressive CD8^+^ T cell activation results in the promotion of tumor progression (Pfister et al., 2021). These results indicate that PD-1 therapy-mediated excessive T cell activation could induce tissue damage and subsequently lead to tumor mutation and progression. Therefore, timely and context-dependent PD-1 therapy is very important for inducing antitumor immunity and preventing side effects.

**TABLE 2:** The therapeutic effects of PD-1 therapy in various immune cells.

Cell types	Therapeutic effects	References
CD8^+^ T cells	Functional restoration	Bally et al. (2016), Hui et al. (2017), Kamphorst et al. (2017b), Khan et al. (2019), Macian et al. (2001), Mathieu et al. (2013), Meng et al. (2018), Oestreich et al. (2008), Staron et al. (2014)
	Promotion of proliferation	
Tconvs	Restoration of cytokine secretion, DC maturation, and cytotoxicity	Balanca et al. (2021), Bronsert et al. (2020), Nagasaki et al. (2020), Oh et al. (2020), Sanchez-Alonso et al. (2020), Shi et al. (2018a), Shi et al. (2018b)
	Restoration of IL-21 expression in Tfhs	
Tregs	Amplification of Treg suppressive function	Kamada et al. (2019), Kumagai et al. (2020)
	Reduction of Treg populations	Lim et al. (2021), Stathopoulou et al. (2018), Yoshida et al. (2020)
	Inhibition of Treg suppressive function and stability	
B cells	Inhibition of IL-10 expression	Wang et al. (2019b), Xiao et al. (2016)
	Restoration of CD8^+^ T cell infiltration and function	
NK cells	Functional restoration	Concha-Benavente et al. (2018), Liu et al. (2017), Quatrini et al. (2021)
ILCs	Enhancement of CD103^+^ DC recruitment into TIME	Moral et al. (2020), Taylor et al. (2017), Wang et al. (2020b)
	Restoration of ILC2 function	
	Promotion of cytokine secretion by ILC3	
TAMs	Promotion of phagocytosis	Dhupkar et al. (2018), Gordon et al. (2017), Kono et al. (2020), Rao et al. (2020)
	Inhibition of M1 to M2 transition	
DCs	Restoration of cytokine secretion, costimulatory molecule expression, antigen presentation, and CD8^+^ T cell function	Karyampudi et al. (2016), Krempski et al. (2011), Lamichhane et al. (2017), Lim et al. (2016)
	Inhibition of T cell activation by blocking *cis* interaction between PD-1 and PD-L1	Zhao et al. (2018)
Myeloid cells	Inhibition of MDSC generation	Strauss et al. (2020)
	Increase the effector myeloid cells	

### 2.4 Resistance to PD-1 Therapy

TI CD8^+^ T cells express PD-1 as well as other ICRs (e.g., TIM3, TIGIT, and LAG3) (Fourcade et al., 2010; Matsuzaki et al., 2010; Anderson et al., 2016). Because other ICRs transduce additional inhibitory signals into TI CD8^+^ T cells, PD-1 therapy could not be effective in enhancing antitumor immunity by reinvigorating the functionality of TI CD8^+^ T cells (Fourcade et al., 2010; Matsuzaki et al., 2010). Furthermore, the expression pattern of immune checkpoint ligands (ICLs) is related to the responsiveness to PD-1 therapy (Lee et al., 2020). Human patients with cancer, who do not express PD-L1 on tumor cells, tend to not respond to PD-1 therapy (Lee et al., 2020). This result suggests that the responsiveness of PD-1 therapy is related to the direct interaction between PD-1 and PD-L1. As mentioned above, human cancer patients with TI PD-1^+^CD28^−^CD8^+^ T cells show resistance to PD-1 therapy (Kamphorst et al., 2017b; Hui et al., 2017; Kim et al., 2021). These TI PD-1^+^CD28^−^CD8^+^ T cells can be reinvigorated by IL-15, indicating that resistance to PD-1 therapy of CD28 deficiency is rescued by IL-15 signaling (Kim et al., 2021). Interestingly, the DNA in exhausted CD8^+^ T cells is highly methylated, indicating that genes related to effector function are inactivated at the transcriptional level (Ghoneim et al., 2017). Because of DNA methylation, TI PD-1^+^CD8^+^ T cells are resistant to PD-1 therapy (Ghoneim et al., 2017). Therefore, targeting DNA methylation in TI CD8^+^ T cells is a promising strategy to overcome resistance to PD-1 therapy.

## 3 CD4^+^Foxp3^-^ Conventional T Cells (Tconvs)

Tconvs play an important role in adaptive immune responses (Zhu and Paul, 2008), but the role of Tconvs in the TIME is considered insignificant in controlling tumors compared to that of CD8^+^ T cells. Recently, it has been discovered that the role of TI Tconvs is also important for antitumor immunity (Quezada et al., 2010; Yamaguchi et al., 2018; Zappasodi et al., 2018; Nagasaki et al., 2020; Balanca et al., 2021; Tay et al., 2021). TI Tconvs play a fundamental role as ‘helper T cells’ that help prime CD8^+^ T cells to kill tumor cells and B cells for antibody production (Yamaguchi et al., 2018; Zappasodi et al., 2018; Balanca et al., 2021; Tay et al., 2021). Additionally, TI Tconvs have a role as “cytotoxic CD4^+^ T cells” that directly kill tumor cells in an MHC II-dependent manner (Quezada et al., 2010; Nagasaki et al., 2020; Tay et al., 2021). Interestingly, some TI Tconvs also express PD-1 (Yamaguchi et al., 2018; Zappasodi et al., 2018; Nagasaki et al., 2020; Balanca et al., 2021) (Table 1).

### 3.1 Expression and Function

As mentioned above, PD-1 is expressed on Tconvs upon TCR stimulation (Agata et al., 1996). The mechanism of PD-1 expression in Tconvs has not been studied as intensively as in CD8^+^ T cells, but is thought to be similar to that in CD8^+^ T cells. Tumor-antigen-specific Tconvs express CD39 and PD-1 (Balanca et al., 2021). These PD-1^+^CD39^+^ Tconvs exhibit a highly exhausted phenotype (Balanca et al., 2021). PD-1 inhibits TI PD-1^+^CD39^+^ Tconv function (e.g., effector cytokine production and dendritic cell (DC) maturation), thereby restraining DC-mediated TI CD8^+^ T cell proliferation (Balanca et al., 2021). This study also identifies that TI PD-1^+^CD39^+^ Tconvs express more TOX and its target genes than TI PD-1^-^ Tconvs (Balanca et al., 2021). In follicular helper T cells (Tfhs), which are responsible for priming B cells to produce neutralizing antibodies (Vinuesa et al., 2016; Crotty, 2019), PD-1 is found to regulate Tfh localization and function in human and mouse tumors (Shi J. et al., 2018; Bronsert et al., 2020; Sanchez-Alonso et al., 2020). Indeed, a high frequency of PD-1^+^ Tfhs correlates with poor prognosis in breast and colorectal tumors (Gu-Trantien et al., 2013; Shi W. et al., 2018; Bronsert et al., 2020). Intriguingly, Tfhs are also found to directly promote TI CD8^+^ T cell effector function by secreting IL-21 in colorectal tumors as well as B cell priming (Shi W. et al., 2018). The expression of IL-21 in TI PD-1^+^ Tfhs is repressed by PD-L1-expressing tumor cells (Shi W. et al., 2018). PD-1 also represses the cytotoxic function of TI Tconvs in MHC II-expressing tumors (Nagasaki et al., 2020; Oh et al., 2020). Collectively, PD-1 expressed on TI Tconvs inhibits effective antitumor immunity by suppressing the functionality of TI Tconvs (Figure 1).

### 3.2 Blockade Effect and Resistance to PD-1 Therapy

It has been identified that PD-1 therapy enhanced antitumor immunity by restoring various TI Tconv functions (Table 2). First, PD-1 therapy restores cytokine production (IFN-γ, TNF-α, IL-2, and IL-12) from TI PD-1^+^CD39^+^ Tconv (Balanca et al., 2021). Additionally, PD-1 therapy enhances TI PD-1^+^CD39^+^ Tconv activity, which differentiates immature DCs into mature DCs, thereby promoting DC-mediated CD8^+^ T cell proliferation (Balanca et al., 2021). In Tfhs, PD-1 therapy restores the expression of IL-21 in TI PD-1^+^ Tfhs (Shi W. et al., 2018). As mentioned above, PD-1 therapy enhances the TI CD8^+^ T cell priming of TI Tfhs in an IL-21-dependent manner (Shi W. et al., 2018). Recently, in a mouse lung tumor model, circulating Tfhs enhance the responsiveness of PD-1 therapy by increasing the number of tertiary lymphoid structures (Sanchez-Alonso et al., 2020). PD-1 therapy also increases cytokine secretion by TI cytotoxic Tconvs (Nagasaki et al., 2020; Oh et al., 2020).

In a mouse tumor model, TI PD-1^+^ cytotoxic Tconvs also express LAG3 (Nagasaki et al., 2020). LAG3 binds to MHC II and transduces inhibitory signals into CD4^+^ T cells (Anderson et al., 2016). Although PD-1 therapy is effective in a mouse tumor model, dual blockade of PD-1 and LAG3 shows a synergistic effect (Nagasaki et al., 2020). This result suggests that PD-1 therapy alone might be insufficient to reinvigorate the functionality of TI Tconvs and that other ICRs could induce resistance to PD-1 therapy.

## 4 CD4^+^Foxp3^+^ Regulatory T Cells (Tregs)

Tregs suppress immune cells and effector T cells for immune homeostasis (Fontenot et al., 2003; Kim et al., 2007; Josefowicz et al., 2012; Campbell, 2015). To reduce antitumor immunity, tumor cells recruit Tregs in a chemokine-dependent manner or provide a favorable environment for Treg proliferation (Campbell, 2015; Son et al., 2020). Accumulated TI Tregs largely reduce antitumor immunity by suppressing effector T cells (Campbell, 2015; Gianchecchi and Fierabracci, 2018; Lucca and Dominguez-Villar, 2020; Son et al., 2020). According to recent studies, a high abundance of TI Tregs and the high level of PD-1 expression in TI Tregs are associated with poor prognosis in various cancer patients (Park et al., 2012; Tanaka and Sakaguchi, 2017; Kim K. H. et al., 2019; Kumagai et al., 2020; Lucca and Dominguez-Villar, 2020). . However, the function of PD-1 in Tregs remains controversial (Table 1).

### 4.1 Expression

Similar with CD8^+^ and Tconvs, Tregs express PD-1 upon TCR stimulation (Agata et al., 1996). TI PD-1^+^ Tregs are observed in various cancer patients and mouse tumor model (Park et al., 2012; Lowther et al., 2016; Kim K. H. et al., 2019; Kamada et al., 2019; Kumagai et al., 2020; Yoshida et al., 2020). A recent study on the TCR repertoire of TI Tregs reveals clues about how TI Tregs express PD-1 in TIME (Ahmadzadeh et al., 2019). Ahmadzadeh *et al.* reveals that TI Tregs exhibit reactivity against tumor antigen and TCR repertoire of TI Tregs is distinct from that of Tconvs in the blood and TIME. This study suggests that TI Tregs are more activated and proliferated in a tumor antigen-selective manner than TI Tconvs, thereby leading to the high level of PD-1 expression in TI Tregs (Ahmadzadeh et al., 2019). Recently, it has been also identified that the expression of PD-1 in TI Tregs is induced by SREBP signaling-induced protein geranylgeranylation (Lim et al., 2021). Collectively, the PD-1 expression in TI Tregs is induced by tumor antigen-specific TCR stimulation and lipid metabolism.

### 4.2 Function

Some groups have suggested that PD-1 represses Treg suppressive function (Lowther et al., 2016; Kamada et al., 2019; Kumagai et al., 2020; Tan et al., 2021). Kamada *et al.* demonstrate that PD-1-deficient Tregs show high suppressive capacity compared to PD-1-intact Tregs in a mouse tumor model. They also suggest that hyperprogression and increased tumor progression after PD-1 therapy in human cancer patients are induced by enhancing Treg function. Mechanistically, PD-1 represses the functionality of TI Tregs by inhibiting the phosphorylation of AKT and ZAP70 (Kumagai et al., 2020). In malignant gliomas, PD-1^+^ Tregs do not suppress effector T cells to the same extent as PD-1^-^ Tregs (Lowther et al., 2016). These PD-1^+^ Tregs show high levels of FoxO1 phosphorylation (Lowther et al., 2016). The level of FoxO1 phosphorylation is increased by PD-1 blockade (Lowther et al., 2016). Taken together, these results suggest that PD-1 on TI Tregs also inhibits TI Treg function (Figure 1). However, there are some debates regarding these results. High levels of FoxO1 phosphorylation are required for the suppressive function of Tregs (Kerdiles et al., 2010; Ouyang et al., 2012; Luo et al., 2016). Inhibition of phosphorylation of AKT signaling is required for the development of functional Tregs (Francisco et al., 2009; Chi, 2012; Lim et al., 2021). Therefore, although these studies suggest that PD-1 inhibits Treg function in the TIME, PD-1 may maintain the stability and functionality of Tregs in the TIME.

In contrast, other studies have suggested the opposite hypothesis that PD-1 promotes the suppressive function of Tregs (Francisco et al., 2009; Park et al., 2012; Park et al., 2015; Asano et al., 2017; Stathopoulou et al., 2018; Kim K. H. et al., 2019; Dong et al., 2020; Yoshida et al., 2020; Lim et al., 2021). PD-1 regulates Treg homeostasis by promoting proliferation and inhibiting apoptosis during low-dose IL-2 therapy (Asano et al., 2017). Additionally, PD-1 is found to maintain Foxp3 expression by inhibiting asparaginyl endopeptidase (AEP) (Stathopoulou et al., 2018). Because Foxp3 is responsible for the suppressive function of Tregs, this hypothesis suggests that PD-1 enhances the functionality of Tregs by maintaining the expression of Foxp3 (Stathopoulou et al., 2018). Lipid metabolism in Treg cells is crucial for the maintenance and functionality of TI Tregs (Wang S. et al., 2020; Lim et al., 2021). Interestingly, PD-1 is associated with the lipid metabolism of TI Tregs (Patsoukis et al., 2015; Lim et al., 2021). Lim *et al.* identify that SREBP signaling is crucial for TI Treg suppressive capacity by upregulating PD-1. Mechanistically, SREBP and PD-1 signaling inhibit the activation of PI3K in TI Tregs, thereby amplifying the suppressive functionality of TI Tregs (Lim et al., 2021). These studies suggest that PD-1 enhances the suppressive function of TI Treg cells (Figure 1). Taken together, the exact function of TI Tregs remains elusive. Therefore, further studies to identify the context-dependent function of TI Tregs would be helpful to understand how PD-1^+^ TI Tregs affect PD-1 therapy.

### 4.3 Blockade Effect

Because the function of PD-1 in Tregs has not been defined precisely, the therapeutic effect of PD-1 therapy on Tregs is also controversial (Table 2). Kamada *et al.* demonstrate that PD-1 therapy enhances the suppressive functionality of TI Tregs in human and mouse models. Kumagai *et al.* identify that the balance in PD-1 expression between TI CD8^+^ T cells and TITregs is crucial for predicting the responsiveness of PD-1 therapy. This study suggests that the preferential consumption of anti-PD-1 antibodies would enhance or reduce antitumor immunity (Kumagai et al., 2020). In contrast, several studies have shown that PD-1 therapy reduces the suppressive function of TI Tregs and enhances antitumor immunity (Stathopoulou et al., 2018; Kim K. H. et al., 2019; Yoshida et al., 2020; Lim et al., 2021). Yoshida *et al.* demonstrate that PD-1 therapy reduces the frequency of TI Tregs in human and mouse osteosarcoma. Additionally, H. R. Kim *et al.* show that PD-1 therapy restrains the functionality of TI Tregs in human and mouse lung cancer. In this regard, PD-1 therapy still enhances antitumor immunity by repressing the functionality and stability of TI Tregs.

### 4.4 Resistance to PD-1 Therapy

Similar to other CD8^+^ T cells and Tconvs, Tregs express other ICRs in the TIME (Wing et al., 2008; Sakuishi et al., 2013; Kurtulus et al., 2015). In Tregs, other ICRs (e.g., CTLA4, TIGIT, and TIM3) are responsible for maintaining the stability and functionality of TI Tregs (Wing et al., 2008; Sakuishi et al., 2013; Kurtulus et al., 2015; Sato et al., 2021). Therefore, there is a possibility that other ICRs still act on the maintenance of Treg stability and functionality in PD-1-blocked TI Tregs, thereby inducing resistance to PD-1 therapy. Several groups, insisting that PD-1 inhibits the suppressive function of TI Tregs, suggest that resistance to PD-1 therapy is induced when PD-1 therapy preferentially acts on TI Tregs rather than TI CD8^+^ T cells (Kamada et al., 2019; Kumagai et al., 2020). As mentioned above, because the function of TI Tregs is controversial, resistance to PD-1 therapy induced by PD-1^+^ TI Tregs needs to be further explored.

## 5 B Cells

It has been shown that TI B cells are associated with responsiveness to PD-1 therapy (Xiao et al., 2016; Guo and Cui, 2019). The role of TI B cells is debatable. Some TI B cells enhance antitumor immunity by producing tumor-specific antibodies, presenting tumor-specific antigens, and secreting cytokines (IFN-γ, TNF-α, and IL-12) (Guo and Cui, 2019). In contrast, other TI B cells, also known as regulatory B cells (Bregs), reduce antitumor immunity by secreting cytokines (IL-10, TGFβ, and IL-35) (Guo and Cui, 2019). The TI B cells express PD-1 (Table 1).

### 5.1 Expression

PD-1 expression in B cells is induced by various factors (Xiao et al., 2016). Using human B cells from healthy blood donors, it is demonstrated that HCC tumor cell culture supernatants increase the number of PD-1^+^ B cells, while normal liver cell culture supernatants do not (Xiao et al., 2016). This result indicates that some factors in tumor cell culture supernatants have the potential to induce PD-1 expression in B cells. Further investigations reveal that CD40 signaling also induces PD-1 expression in B cells (Xiao et al., 2016). BCL6 is upregulated in PD-1^+^ B cells and related to PD-1 expression (Xiao et al., 2016). Using inhibitors of various signaling pathways, JNK, p38, and NF-κB contribute to the induction of PD-1 expression by upregulating BCL6 expression (Xiao et al., 2016). Several cytokines (including IL-1β, IL-6, and IL-10) do not induce PD-1 expression (Xiao et al., 2016). Notably, IL-4 represses CD40 signaling-dependent PD-1 expression in B cells (Xiao et al., 2016). Additionally, phosphorylation of STAT6 is linked to IL-4 stimulation and is repressed in PD-1^+^ B cells. (Xiao et al., 2016).

### 5.2 Function

PD-1 induces immunosuppressive IL-10 expression in TI B cells from patients with HCC (Xiao et al., 2016). In conventional B cells, the TLR4 agonist, CD40 signaling, and anti-IgM addition can induce IL-10 expression (Xiao et al., 2016). However, in TI B cells, these factors do not induce IL-10 expression, and only PD-1 signaling can induce IL-10 expression (Xiao et al., 2016). IL-10 secreted by TI PD-1^+^ T cells consequently suppresses CD8^+^ T cell infiltration and function, thereby inhibiting effective antitumor immunity (Xiao et al., 2016). Furthermore, in a mouse HCC model, anti-IL-10R administration delays tumor growth by reinvigorating CD8^+^ T cell infiltration and function to a similar extent as anti-PD-L1 administration (Xiao et al., 2016). This result suggest that PD-1 expressed on TI B cells mediates T cell suppression and results in rapid tumor growth. However, in patients with differentiated thyroid cancer, PD-1 signaling do not induce IL-10 expression in TI B cells (Wang et al., 2019b). This result indicates that PD-1 function in TI B cells can be context-dependent. This study identifies that TI PD-1^+^ B cells result in impairment of T cell proliferation in a PD-L1-dependent manner (Wang et al., 2019b), which suggests that TI PD-1^+^ B cells control antitumor immunity by directly suppressing T cell proliferation. Taken together, although PD-1 function in TI B cells is context-dependent, TI PD-1^+^ B cells mediate T cell suppression and induce rapid tumor growth (Figure 1).

### 5.3 Blockade Effect

PD-1 therapy represses IL-10 expression in TI B cells from patients with HCC, suggesting that PD-1 therapy can enhance antitumor immunity by impairing the TI PD-1^+^ B cell suppressive capacity (Xiao et al., 2016) (Table 2). Using a mouse hepatoma model, PD-1 therapy delays tumor growth by recovering CD8^+^ T cell infiltration and function in a TI PD-1^+^ B cell-dependent manner (Xiao et al., 2016) (Table 2). Additionally, Wang *et al.* demonstrate that PD-1 therapy increases T cell viability (Wang et al., 2019b) (Table 2). However, this result is limited as this effect is observed only *in vitro*, and there is no *in vivo* evidence. Therefore, further studies on the direct and *in vivo* effects of PD-1 therapy on TI PD-1^+^ B cells are needed.

## 6 Natural Killer Cells

NK cells play a critical role in antitumor immunity by directly killing tumor cells such as CD8^+^ T cells (Shimasaki et al., 2020). Tumor cells downregulate MHC on their surface to escape recognition by CD8^+^ T cells (Shimasaki et al., 2020). However, NK cells recognize MHC deficiency in tumor cells and kill MHC-deficient tumor cells in a cytokine-dependent manner. TI NK cells also express PD-1, and TI PD-1^+^ NK cells are suppressed by the engagement of PD-L1 expressed on tumor cells or other immune cells (Zhang and Liu, 2020) (Table 1).

### 6.1 Expression

Recently, glucocorticoids (GCs) and various cytokines (including IL-12, IL-15, and IL-18) induce the expression of PD-1 in TI NK cells (Quatrini et al., 2021). GCs are steroid hormones that have an immunosuppressive effect. As the GC receptor is expressed on every cell type, NK cells are also affected by the immune-suppressive effect of GCs. One of the immunosuppressive effects of GCs is the induction of PD-1 expression on NK cells (Quatrini et al., 2021). These factors preferentially affect CD56^bright^ NK cells and induce PD-1 expression (Quatrini et al., 2021). Mechanistically, in CD56^bright^ NK cells, GCs upregulate the expression of PD-1 by promoting a transcriptional program related to translation (Quatrini et al., 2021). In patients with head and neck cancer, cetuximab (anti-EGFR) treatment induces NK cell activation, thereby increasing the frequency of PD-1^+^ NK cells (Concha-Benavente et al., 2018). These results suggest that NK cells would express PD-1 during activation. The specific mechanism of PD-1 expression in NK cells needs to be further elucidated.

### 6.2 Function

In various tumor types, TI PD-1^+^ NK cells exhibit less functional phenotypes (Liu et al., 2017; Concha-Benavente et al., 2018; Vari et al., 2018; Yin et al., 2018; Trefny et al., 2020). Liu *et al.* demonstrate that PD-1 regulates NK cell function by suppressing the activation of PI3K/AKT signaling in NK cells. Additionally, PD-1^+^ NK cells exhibit impaired cytotoxicity against PD-L1-expressing tumor cells (Quatrini et al., 2021). In patients with head and neck cancer, cetuximab-activated PD-1^+^ NK cells are functionally repressed by PD-L1-expressing tumor cells (Concha-Benavente et al., 2018). Taken together, PD-1 suppresses the effector function of NK cells (Figure 1).

### 6.3 Blockade Effect

TI PD-1^+^ NK cells can be reinvigorated by PD-1 therapy (Concha-Benavente et al., 2018; Trefny et al., 2020; Vari et al., 2018) (Table 2). In Hodgkin lymphoma, PD-1^+^CD3^−^CD56^hi^CD16^negative^ NK cells are repressed by PD-L1-expressing monocytes (Vari et al., 2018). These PD-1^+^CD3^−^CD56^hi^CD16^negative^ NK cells are reinvigorated by depletion of PD-L1-expressing monocytes or PD-1 therapy (Vari et al., 2018). In patients with head and neck cancer, PD-1 therapy enhances the functionality of cetuximab-activated PD-1^+^NK cells by inhibiting interactions with PD-L1-expressing tumor cells (Concha-Benavente et al., 2018). Collectively, in various tumor types, PD-1 therapy enhances antitumor immunity by reinvigorating NK cell function.

### 6.4 Resistance to PD-1 Therapy

Similar to T cells, TI PD-1^+^ NK cells express other ICRs (Seo et al., 2018; Yin et al., 2018; Zhang et al., 2018). Although PD-1 therapy blocks the interaction between PD-1 and PD-L1, other ICRs on TI NK cells suppress the functionality of TI NK cells. Multiple expression of ICRs in NK cells results in resistance to PD-1 therapy. Therefore, blocking multiple ICRs effectively restores the functionality of TI NK cells, thereby overcoming resistance to PD-1 therapy (Seo et al., 2018; Yin et al., 2018; Zhang et al., 2018). Seo *et al.* demonstrate that intratumoral administration of IL-21 enhances the efficacy of PD-1/TIM3 therapy by recruiting NK cells into the TIME in a CXCR3-dependent manner. They also demonstrate that IL-21 cytokine therapy have a synergistic effect with PD-1/TIM3 therapy in human and mouse tumors (Seo et al., 2018). In particular, in various human cancer patient samples (including colon cancer, bladder cancer, and melanoma), a combination of IL-21 administration and PD-1/TIM3 therapy reinvigorate the functionality of PD-1^+^TIM3^+^ NK cells and overcome resistance to PD-1/TIM3 therapy (Seo et al., 2018). Therefore, IL-21 administration can be a good target for overcoming resistance to NK cell-dependent PD-1 therapy.

## 7 Innate Lymphoid Cells

ILCs are derived from common lymphoid progenitors and are mostly found in tissues (Vivier et al., 2018). ILCs are responsible for remodeling and repairing tissues, lymphoid organogenesis, and innate immune responses against pathogens and tumors (Vivier et al., 2018; Mariotti et al., 2019). ILCs are classified as ILC1, ILC2, and ILC3 (Vivier et al., 2018; Mariotti et al., 2019). ILC1, ILC2, and ILC3 are similar to CD4^+^ T helper (Th) 1, Th2, and Th17 cells, respectively (Vivier et al., 2018; Pesce et al., 2020). ILCs also express PD-1. It has been identified that PD-1 on ILCs plays a distinct role in regulating antitumor immunity (Mallett et al., 2019) (Table 1).

### 7.1 Expression

PD-1 expression in ILC1 has not yet been found. In a mouse model, it is first found that PD-1 is expressed in KLRG1^+^ILC2 (Taylor et al., 2017). In KLRG1^+^ILC2, PD-1 expression is induced through stimulation with IL-2, IL-7, and IL-33 (Taylor et al., 2017). Because this study does not use a mouse tumor model, we do not identify whether PD-1 regulation in ILC2 is also observed in mouse tumor tissues. Recently, in patients with human colorectal and pancreatic cancer, ILC2 expresses PD-1 (Wang H. et al., 2020; Moral et al., 2020). Mechanistically, IL-33/ST2 signaling induces PD-1 expression in TI ILC2 (Moral et al., 2020). In pleural effusions of various patients with cancer (mesothelioma and adenocarcinoma), PD-1 is also expressed in ILC3 (Tumino et al., 2019). However, the mechanism by which PD-1 expression in ILC3 is induced is yet to be identified (Tumino et al., 2019). Therefore, PD-1 expression in ILC1 and the specific mechanism of PD-1 expression in ILC3 need to be investigated for effective PD-1 therapy.

### 7.2 Function

TI ILC2 enhances antitumor immunity indirectly (Moral et al., 2020). Recombinant IL-33 (rIL-33)-activated TI ILC2 secretes CCL5, which recruits CD103^+^ dendritic cells (DCs) (Moral et al., 2020). Because CD103^+^ DCs are responsible for activating CD8^+^ T cells, rIL-33-activated TI ILC2 enhances antitumor immunity by inducing CD103^+^ DC-mediated TI CD8^+^ T cell activation (Moral et al., 2020). Additionally, rIL-33-activated TI ILC2 expresses PD-1 (Moral et al., 2020). In fact, adoptive transfer of rIL-33-activated TI PD-1^+^ ILC2 into ILC2-deficient mice controls tumor progression, indicating that rIL-33-activated TI PD-1^+^ ILC2 is functional and enhances antitumor immunity (Moral et al., 2020). Notably, adoptive transfer of rIL-33-activated TI PD-1^−/−^ ILC2 into ILC2-deficient mice enhances antitumor immunity more than that of rIL-33-activated TI PD-1^+^ ICL2 into ILC2-deficient mice, suggesting that PD-1 signaling restrains the optimal functionality of rIL-33-activated TI ILC2 (Moral et al., 2020). They also demonstrate that blocking PD-1 on TI ILC2 enhances antitumor immunity in an rIL-33-activated TI ILC2-transferred mouse tumor model (Moral et al., 2020). This result indicates that PD-1 signaling suppresses the functionality of TI ILC2. However, they do not show whether blocking PD-1 on TI ILC2 affects CCL5 expression in TI ILC2. Therefore, further studies on the direct relationship between CCL5 expression and PD-1 signaling in TI ILC2 are required. In ILC3, PD-1 inhibits cytokine production (IFN-γ and TNF-α) (Tumino et al., 2019). Collectively, PD-1 reduces antitumor immunity by inhibiting the ILC effector function (Figure 1).

### 7.3 Blockade Effect

Because PD-1 signaling suppresses the functionality of TI ILC2, ablation or blockade of PD-1 on TI ILC2 resultes in improved functionality of TI ILC2 and enhances antitumor immunity in a mouse pancreatic tumor model (Moral et al., 2020) (Table 2). As mentioned above, although TI ILC2 enhances antitumor immunity by recruiting CD103^+^ DCs in a CCL5-dependent manner (Moral et al., 2020), it is still unclear whether PD-1 therapy affects the expression of CCL5 in TI ILC2. Nevertheless, the combination of PD-1 therapy and rIL-33 treatment effectively controls tumor progression by inducing TI ILC2-dependent CD103^+^ DC migration into the TIME (Moral et al., 2020). In TI ILC3, PD-1 therapy enhances antitumor immunity by inducing cytokine secretion (IFN-γ and TNF-α) by TI ILC3 (Tumino et al., 2019) (Table 2). Taken together, PD-1 therapy enhances antitumor immunity by augmenting the functionality of TI ILCs.

### 7.4 Resistance to PD-1 Therapy

PD-1 therapy alone is not sufficient to enhance antitumor immunity in a mouse pancreatic tumor model (Moral et al., 2020). The combination of PD-1 therapy and rIL-33 administration enhances antitumor immunity in an ILC2-dependent manner (Moral et al., 2020). This result indicates that PD-1 therapy is dependent on the activation state of TI ILC2 in a mouse pancreatic tumor model and that TI IL2 activation by IL-33 signaling can overcome resistance to PD-1 therapy (Moral et al., 2020). Meanwhile, resistance to PD-1 therapy triggered by PD-1^+^ ILC3 is poorly understood.

## 8 Tumor-Associated Macrophages

There are two types of TAMs: pro-inflammatory M1 and anti-inflammatory M2 TAMs (Noy and Pollard, 2014; Roszer, 2015). Pro-inflammatory M1 TAMs promote phagocytosis of tumor cells and anti-inflammatory M2 TAMs secrete immunosuppressive cytokines (e.g., IL-10, IL-6, and TGFβ) (Noy and Pollard, 2014; Roszer, 2015; Gordon et al., 2017). These TAMs also express PD-1 (Table 1).

### 8.1 Expression

Macrophages express PD-1 in response to type I interferon (IFN) (Cho et al., 2008). Unlike PD-1 on T cells, PD-1 on macrophages is induced by the transcription factor nuclear factor-kappa B (NF-κB) upon TLR2/4 stimulation, but not by MAP kinase (Bally et al., 2015). PD-1 is also expressed in both human and mouse TAMs (Cho et al., 2008; Bally et al., 2015; Gordon et al., 2017). However, the mechanism of tumor-specific PD-1 expression is unclear. Two different groups recently reveal the mechanism by which PD-1 expression is regulated in TAMs (Lyle et al., 2019; Tartey et al., 2021). Lyle *et al.* explain that PD-1 in TAMs is downregulated by casitas B lymphoma (c-Cbl) E3 ubiquitin ligase in colorectal cancer. TAMs of c-Cbl knockout (c-Cbl^+/-^) mice express more PD-1 than those of wild type mice and exhibit a reduction in phagocytosis (Lyle et al., 2019). Mechanistically, c-Cbl binds to the cytosolic tail of PD-1 and downregulates PD-1 by ubiquitination-proteasomal degradation (Lyle et al., 2019). Another group demonstrates that the MyD88/IL1 receptor (IL1R) axis plays an important role in regulating the expression of PD-1 in TAMs (Tartey et al., 2021). The MyD88/IL1R axis in TAM recruits transcription factor nuclear factor-kappa B (NF-κB) on the PD-1 promoter, thereby upregulating the expression of PD-1 on TAMs (Tartey et al., 2021).

### 8.2 Function

PD-1 expressed on M1 TAMs reduces antitumor immunity by inhibiting phagocytosis (Gordon et al., 2017; Kono et al., 2020). Additionally, PD-1 induces the M1 to M2 transition (Gordon et al., 2017; Dhupkar et al., 2018; Rao et al., 2020). PD-1 is also involved in the differentiation of TAMs (Gok Yavuz et al., 2019). Cancer-associated fibroblasts (CAFs) resident in the TIME recruit monocytes by monocyte chemotactic protein-1 (MCP-1) and stromal cell-derived factor-1 (SDF-1) (Gok Yavuz et al., 2019). The recruited monocytes are differentiated into M2 TAMs by CAFs and express more PD-1 than the normal fibroblast (NF)-educated monocytes (Gok Yavuz et al., 2019). These CAF-educated monocytes exhibit a more suppressive phenotype on the PD-1 axis than NF-educated monocytes (Gok Yavuz et al., 2019). Tissue samples from human breast cancer patients show that a higher frequency of CAFs is related to the abundance of TAMs (Gok Yavuz et al., 2019). Collectively, PD-1 is involved in suppressing the phagocytosis of M1 TAMs and inducing the differentiation of M2 TAMs (Figure 1).

### 8.3 Blockade Effect

The abundance of PD-1-expressing TAMs correlates with poor prognosis in human cancer patients (Chen et al., 2020; Gordon et al., 2017; Kono et al., 2020). In a mouse model, PD-1 therapy reduces tumor growth (Gordon et al., 2017) (Table 2). This effect is abolished by TAM depletion, indicating that PD-1 therapy enhances antitumor immunity by amplifying TAM phagocytosis (Gordon et al., 2017) (Table 2). In patients with osteosarcoma, PD-1 therapy inhibits M1 to M2 transition and increases the frequency of M1 TAMs, thereby enhancing antitumor immunity (Dhupkar et al., 2018) (Table 2). This effect has also been observed in patients with glioblastoma (Rao et al., 2020). This study confirms the TAM-mediated PD-1 therapy effect by capitalizing on CD8^+^ T cell-deficient mice (Rao et al., 2020).

## 9 Dendritic Cells

DCs are professional antigen-presenting cells that are responsible for priming and activating T cells (Wculek et al., 2020). In the TIME, the tumor-antigen uptake ability of DCs is important for the expansion of tumor-antigen-specific T cells (Wculek et al., 2020). Tumor cells inhibit DC migration into the TIME by secreting CCL4 (Spranger et al., 2015). Inhibition of DC infiltration promotes tumor progression by deteriorating T cell priming (Spranger et al., 2015; Salmon et al., 2016). DCs usually express PD-L1 (Salmon et al., 2016; Wculek et al., 2020). PD-L1^+^ DCs show reduced T cell priming ability, and PD-L1 blockade increases T cell priming (Salmon et al., 2016). A high frequency of PD-L1^+^ DCs in the TIME is associated with a poor prognosis in human cancer patients (Mu et al., 2011; Wculek et al., 2020). Interestingly, DCs are found to express both PD-1 and PD-L1 (Table 1).

### 9.1 Expression

The mechanism of PD-1 expression in DCs has not yet been elucidated. In a mouse ovarian tumor model, PD-1 expression on TI DCs is demonstrated to be regulated by IL-10 cytokine (Lamichhane et al., 2017). Mechanistically, IL-10 treatment of mouse bone marrow-derived DCs (BMDCs) induces PD-1 expression in a STAT3-dependent manner (Lamichhane et al., 2017). Except for IL-10, other mechanisms of PD-1 expression in TI-DCs remain elusive. Further studies are needed to identify the mechanism of PD-1 expression in TI-DCs.

### 9.2 Function

PD-1 expressed on DCs is associated with immune suppression (Krempski et al., 2011; Karyampudi et al., 2016; Lim et al., 2016; Lamichhane et al., 2017; Zhao et al., 2018). In human and mouse hepatocellular carcinoma, PD-1-expressing CD11c^+^ TI DCs have been identified and have the suppressive capacity to repress CD8^+^ T cell function (Lim et al., 2016). This study demonstrates that PD-1-deficient DCs are defective in suppressing CD8^+^ T cell function, thereby enhancing antitumor immunity (Lim et al., 2016). In a mouse ovarian tumor model, PD-1^+^ DCs accumulate in the TIME and suppress T cell function and infiltration (Krempski et al., 2011; Karyampudi et al., 2016; Lamichhane et al., 2017). PD-1 on TI DCs from human ovarian cancer patients and mouse tumor tissues suppresses cytokine production (TNF-α and IL-6) and costimulatory molecule expression (CD40 and CD80) (Karyampudi et al., 2016). Mechanistically, PD-1 mainly regulates the NF-κB pathway (Krempski et al., 2011; Karyampudi et al., 2016). PD-1 represses cytokine secretion and costimulatory molecule expression in TI DCs by preventing IκBα degradation, indicating that maintenance of IκBα restricts NF-κB subunit p65 into the cytosol (Karyampudi et al., 2016). Furthermore, PD-1 also retrains antigen presentation and MHC I expression of TI DCs in an NF-κB-dependent manner (2016 Cancer research). Notably, PD-1 on DCs interacts with PD-L1 expressed on themselves (Zhao et al., 2018). This *cis* interaction leads to the PD-L1 blocking effect of DC itself, inducing T cell activation (Zhao et al., 2018). This result is in contrast to the already known suppressive function of PD-1 on DCs. However, the *in vivo* role of the *cis* interaction between PD-1 and PD-L1 on DCs has not been identified (Zhao et al., 2018). Therefore, it needs to be determined whether this *cis* function of PD-1 in DCs works in a mouse tumor model. Collectively, except for the cis function of PD-1 in DCs, PD-1 mainly controls various DC characteristics (e.g., cytokine secretion, antigen presentation, costimulatory molecule expression, and MHC I expression) (Figure 1).

### 9.3 Blockade Effect

PD-1 therapy increases DC function and enhances T cell immunity (Karyampudi et al., 2016; Krempski et al., 2011; Lamichhane et al., 2017) (Table 2). Cytokine secretion, costimulatory molecule expression, antigen presentation, and MHC I expression in TI DCs are increased by PD-1 therapy, indicating that PD-1 therapy enhances antitumor immunity by restoring DC function and DC-mediated T cell activation (Krempski et al., 2011; Karyampudi et al., 2016). Mechanistically, PD-1 therapy-induced translocation of the NF-κB subunit p65 into the nucleus activates the NF-κB target genes by degrading IκBα in TI DCs (Karyampudi et al., 2016). Interestingly, PD-1 therapy increases IL-10 expression in TI-DCs (Lamichhane et al., 2017). As mentioned above, because IL-10 induces PD-1 expression on TI DCs, PD-1 blocking and IL-10 expression form a feedback loop (Lamichhane et al., 2017). This result suggest that this feedback loop in TI DCs maintains a suppressive environment and consequently results in resistance to PD-1 therapy (Lamichhane et al., 2017). Additionally, PD-1 therapy can inhibit T cell activation by preventing *cis* interactions with PD-L1 expressed on DCs (Zhao et al., 2018). However, the *in vivo* role of the *cis* interactions has not been verified (Zhao et al., 2018).

### 9.4 Resistance to PD-1 Therapy

As mentioned above, IL-10 expression in TI-DCs is induced by PD-1 therapy (Lamichhane et al., 2017). Consequently, increased IL-10 expression by PD-1 therapy maintains suppressive TIME, inducing resistance to PD-1 therapy (Lamichhane et al., 2017). Therefore, a combination therapy of PD-1 therapy and IL-10 neutralization makes resistant tumors sensitive to PD-1 therapy in a mouse ovarian tumor model (Lamichhane et al., 2017). This result suggests that DC-dependent resistance to PD-1 therapy can be overcome by IL-10 neutralization.

## 10 Myeloid Cells

### 10.1 Expression

TI myeloid cells express PD-1 (Strauss et al., 2020) (Table 1). Among myeloid cells, PD-1 on granulocyte/macrophage progenitors (GMPs) plays an important role in regulating their differentiation into myeloid-derived suppressive cells (MDSCs) during emergency myelopoiesis, which is the cellular proliferation induced by immunologic stress (Strauss et al., 2020). GMPs slightly express PD-1 in the naïve state and the expression of PD-1 is induced in the context of the tumor or in response to several factors such as granulocyte colony-stimulating factor (G-CSF), granulocyte-macrophage colony growth factor (GM-CSF), and TLR4 agonist (Strauss et al., 2020).

### 10.2 Function

Specific ablation of PD-1 on myeloid cells reduces the populations of GMPs and MDSCs and increases the population of effector immune cells, thereby enhancing antitumor immunity (Strauss et al., 2020). The functionality of T cells cocultured with PD-1-deficient myeloid cells is enhanced compared to that cocultured with PD-1-intact myeloid cells, indicating that PD-1-deficient myeloid cells are less suppressive than the PD-1-intact myeloid cells (Strauss et al., 2020). Mechanistically, PD-1-deficient GMPs increase the activation of ERK1/2, mTORC1, and STAT1 during emergency myelopoiesis (Strauss et al., 2020). Inactivation of these signaling pathways is crucial for MDSC generation. Therefore, PD-1 signaling is responsible for MDSC generation by inhibiting the activation of ERK1/2, mTORC1, and STAT1 signaling (Strauss et al., 2020). PD-1 also regulates the metabolism of myeloid cells (Strauss et al., 2020). PD-1-deficient GMPs increase the metabolites of glycolysis, pentose phosphate pathway, and TCA cycle compared to PD-1-intact GMPs (Strauss et al., 2020). Notably, PD-1-deficient GMPs show enhanced cholesterol synthesis (Strauss et al., 2020). Since cholesterol synthesis is responsible for the differentiation of pro-inflammatory myeloid cells, PD-1 signaling in GMPs is crucial for accumulating MDSCs by repressing cholesterol synthesis (Strauss et al., 2020) (Figure 1).

### 10.3 Blockade Effect

This study demonstrate that PD-1 therapy enhanced antitumor immunity by inhibiting the generation of MDSCs from GMPs and increasing the effector myeloid cells in immunocompromised mice (Rag2^−/−^) (Strauss et al., 2020) (Table 2). Additionally, because PD-1 on myeloid cells is expressed in the early phase during tumor-mediated emergency myelopoiesis, PD-1 therapy for early-stage tumors will be optimal to enhance antitumor immunity in a myeloid-dependent manner (Strauss et al., 2020). Because PD-1 expressed on myeloid cells is recently identified, the myeloid cell-dependent PD-1 therapy effect is still poorly understood. Therefore, further identification of PD-1 function in myeloid cells will help to understand the mechanism of PD-1 therapy.

## 11 Tumor Cells

Tumor cells usually express PD-L1 on their surface and tumor-expressing PD-L1 has been known to be a representative biomarker to predict a response to PD-1 blockade (Lee et al., 2020). However, immunohistochemical detection of PD-L1 from tumor biopsy samples does not often reflect the entire characteristics of TIME due to its heterogeneity (Guibert et al., 2018). Since circulating tumor cells (CTCs), which are disseminated cancer cells in circulation, are easily obtained from the blood without surgery (Guibert et al., 2018; Bergmann et al., 2020; Winograd et al., 2020) and reflect the characteristics of TIME better than a biopsy (Lin et al., 2018), CTCs is gradually attracting attention as a real-time biomarker in various cancer patients with metastatic and therapy-resistant disease (Mazel et al., 2015; Kloten et al., 2019; Bergmann et al., 2020; Liu et al., 2020; Winograd et al., 2020). Actually, PD-L1 expression on CTCs are associated with poor prognosis in various cancer patients (Mazel et al., 2015; Kloten et al., 2019; Bergmann et al., 2020; Liu et al., 2020; Winograd et al., 2020). Therefore, additional studies on PD-L1 expression in CTCs and its possibility to predict anti-PD-1 therapy response would be needed.

Of interest, it has been reported that various types of cancer cells occasionally express PD-1, even though the underlying mechanism and function of tumor cell-expressing PD-1 have not been clearly studied.

### 11.1 Expression

PD-1 is expressed not only in immune cells, but also in tumor cells. However, the mechanism of PD-1 expression in tumor cells remains controversial (Table 3).

**TABLE 3:** PD-1 expressed on tumor cells.

Cancer type	Tumor cell-intrinsic PD-1 function	Mechanism	References
Melanoma	PD-1 promoted tumor growth	PD-1 increased the level of phosphorylation of S6, mTOR components, and eIF4E	Kleffel et al. (2015), Schatton et al. (2010)
Hepatoma		PD-1 increased the level of phosphorylation of S6, mTOR components, and eIF4E	Li et al. (2017)
Pancreatic cancer		PD-1 activated the hippo pathway and increased the expression of CYR61 and CTGF.	Pu et al. (2019)
NSCLC	PD-1 inhibited tumor growth	Mechanism was not specified	Du et al. (2018)
Lung cancer		PD-1 inhibited the activation of AKT and ERK signaling	Wang et al. (2020c)

mTOR, mammalian target of rapamycin; eIF4E, eukaryotic initiation factor 4E; CYR61, cysteine-rich angiogenic inducer 61; CTGF, connective tissue growth factor; NSCLC, non-small-cell lung carcinoma; ERK, extracellular signal-regulated kinase.

### 11.2 Function

The function of tumor cell-intrinsic PD-1 is controversial. In this review, we introduce both the oncogenic and tumor-suppressive functions of PD-1 in tumor cells (Figure 1). Several groups have suggested that PD-1 enhances tumor growth (Schatton et al., 2010; Kleffel et al., 2015; Li et al., 2017; Pu et al., 2019). They demonstrate that PD-1 expressed on tumor cells increases the level of phosphorylation of S6 (pS6), mTOR effector molecules, and eukaryotic initiation factor 4E (eIF4E), which are responsible for cellular proliferation (Schatton et al., 2010; Kleffel et al., 2015; Li et al., 2017). Mutations in the immunoreceptor tyrosine-based inhibitory motif (ITIM, Y225F) and the immunoreceptor tyrosine-based switch motif (ITSM, Y248F) in the cytosolic tail of PD-1 expressed on melanoma cells decrease tumor growth, indicating that these ITIM and ITSM in PD-1 have an important role in regulating tumor cell-intrinsic PD-1-mediated tumorigenesis (Kleffel et al., 2015). Analysis of biopsies from human patients with advanced-stage melanoma who received PD-1 therapy reveal that reduced pS6 in tumor cells after PD-1 therapy positively correlates with responsiveness to PD-1 therapy and enhances overall survival (Kleffel et al., 2015). Additionally, PD-1 in tumor cells activates the Hippo pathway (specifically AYR61/CTGF), thereby enhancing tumor cell proliferation (Pu et al., 2019).

However, in lung tumors, other groups have suggested that PD-1 expressed on tumor cells inhibits their proliferation and that PD-1 blockade enhances tumor growth by activating AKT and ERK1/2 (Du et al., 2018; Wang X. et al., 2020). They demonstrate that the knockdown of PD-1 in lung tumor cells increases cell proliferation by upregulating the phosphorylation levels of AKT and ERK1/2, but not S6 (Du et al., 2018; Wang X. et al., 2020). In this study, the mutations of ITIM and ITSM, which is completely identical to the mutation mentioned above, result in the enhancement of tumor cell proliferation by activating AKT and ERK signaling (Wang X. et al., 2020). Additionally, this study demonstrates that SHP2 is not responsible for the function of PD-1 in tumor cells, unlike T cells (Wang X. et al., 2020).

### 11.3 Blockade Effect and Resistance to PD-1 Therapy

Because the function of PD-1 in tumor cells is also controversial, as in Tregs, the effect of tumor cell-specific PD-1 therapy is also elusive (Table 4). In the light of the oncogenic function of PD-1, PD-1 therapy is effective in delaying tumor growth in immunocompromised mice (Kleffel et al., 2015; Li et al., 2017; Pu et al., 2019). This result indicates that PD-1 therapy can directly affect tumor cells by suppressing their proliferation. In contrast, in the light of the tumor-suppressive function of PD-1, PD-1 therapy increases tumor progression in immunocompromised mice (Wang X. et al., 2020). According to these results, PD-1-expressing tumor cells can induce resistance to PD-1 therapy.

**TABLE 4:** The therapeutic effects of PD-1 therapy in tumor cells.

Cancer type	Therapeutic effects	References
Melanoma	Inhibition of tumor growth	Kleffel et al. (2015), Schatton et al. (2010)
Hepatoma		Li et al. (2017)
Pancreatic cancer		Pu et al. (2019)
NSCLC	Promotion of tumor growth	Du et al. (2018)
Lung cancer		Wang et al. (2020c)

Several groups have argued that PD-1 is expressed in tumor cells. Because the characteristics of tumor cells are heterogeneous and determined by their origin, the function of PD-1 expressed on tumor cells can differ depending on the tumor type. Therefore, it is necessary to further identify the distinct functions of PD-1 depending on the tumor type.

### 12 Perspectives

PD-1 therapy is effective in reinvigorating the functionality of CD8^+^ T cells, thereby enhancing antitumor immunity (McLane et al., 2019). However, about 70% of cancer patients fail to respond to PD-1 therapy (Emens et al., 2017). Various clinical trials and studies have been conducted to improve the responsiveness to PD-1 therapy by identifying the characteristics of PD-1^+^CD8^+^ T cells (Kamphorst et al., 2017b; Kim H. R. et al., 2019; Khan et al., 2019). Interestingly, the expression, function, and therapeutic effect of PD-1 in other immune and tumor cells have been recently reported. The overall understanding of PD-1 expressed on various immune cells and tumor cells will be important for elucidating the mechanisms of PD-1 therapy. In this review, we introduced various PD-1 functions in TI immune cells. Given that PD-1 largely inhibits effector functions that delay tumor growth and kill tumor cells, PD-1 therapy mainly enhances antitumor immunity by functional restoration of effector immune cells. However, PD-1 function is still debatable in some suppressive immune and tumor cells.

TI B cells are also one of the debatable populations and their function in the TIME is controversial. While the therapeutic effect of PD-1 therapy on TI Bregs is well identified (Xiao et al., 2016), that on TI B cells responsible for producing a tumor-specific antibody, tumor-antigen presentation, and secretion of cytokines is poorly understood. In addition, PD-1 expression in these TI B cells is not identified. Therefore, further studies are needed to identify PD-1 expression and function in these TI B cells. PD-1 function in tumor cells is also unclear (Schatton et al., 2010; Kleffel et al., 2015; Li et al., 2017; Du et al., 2018; Pu et al., 2019; Wang X. et al., 2020). The incidence of tumors is induced by various factors (e.g. somatic mutations, environmental factors, etc). The characteristics of tumor cells are heterogeneous according to the tumor type. This heterogeneity can be one of the reasons why PD-1 acts differently on tumor cell types. Since the majority of TIME is composed of tumor cells, it is necessary to accurately identify the expression and function of PD-1 in tumor cells for exact evaluating the therapeutic effect of PD-1 therapy.

Interestingly, PD-1 may act differently in Tconvs and Tregs. PD-1 inhibits various TCR downstream signaling pathways in T cells. Among the various TCR downstream signaling pathways, the mTOR pathway is a well-established downstream signaling pathway of TCRs and is known to play different roles in Tconvs and Tregs. The mTOR pathway in T cells is highly downregulated by several inhibitory molecules (e.g., PTEN, TSC1, and LKB1) under steady-state conditions (Chi, 2012). During TCR engagement, the mTOR pathway is activated for the differentiation of naïve CD4^+^ T cells into T helper cell effector lineages, while mTOR activation suppresses Treg differentiation (Chi, 2012). Indeed, PD-1 has been demonstrated to inactivate the mTOR pathway via the dephosphorylation of mTOR components and to stabilize Treg development (Francisco et al., 2009), suggesting that PD-1 inhibits Tconvs and amplifies Tregs. Although there is still an opposing suggestion that PD-1 inhibits Treg function, in terms of regulation of the TCR downstream signaling pathway by PD-1, the suggestion that PD-1 induces the function and development of Tregs also makes sense. Therefore, the exact function of PD-1 in Tregs should be further investigated in various contexts.

TIME is a complex and diverse environment and these complexity and diversity can influence on the responsiveness to PD-1 therapy (Binnewies et al., 2018). As analyzing these complexity and diversity, several reliable biomarkers has been explored to predict the responsiveness to PD-1 therapy and select cancer patients who successfully respond to PD-1 therapy (Kamphorst et al., 2017a; Binnewies et al., 2018; Khan et al., 2019; Lee et al., 2020). One of the biomarkers discovered is the examination of PD-1 expression in TI CD8^+^ T cells (Kamphorst et al., 2017a). In the same manner, since the role of PD-1 has been reported in various TI immune cells and tumor cells, an entire examination of PD-1 expression in TIME can predict which cell are dominantly targeted by PD-1 therapy and the therapeutic effect of PD-1 therapy induced by certain cells. Additionally, this examination can suggest a promising strategy to overcome resistance to PD-1 therapy if cancer patients do not respond to PD-1 therapy. Therefore, identification of PD-1 function is important for understanding the mechanisms underlying various immune cell-dependent effects of PD-1 therapy. This review summarizes PD-1 function in TI immune cells and tumor cells and provides insights into the comprehensive mechanism underlying the therapeutic effect of PD-1 therapy.
